# A Review of Zero Trust Architecture: Principles, Applications, and Implementation Challenges in Communication, Navigation, and Surveillance (CNS) Systems

**DOI:** 10.3390/s26123813

**Published:** 2026-06-15

**Authors:** Nompilo Ngema, Bakhe Nleya, Rito Clifford Maswanganyi

**Affiliations:** Department of Computer and Electronic Engineering, Durban University of Technology, Durban 4001, South Africa; nompilongema@gmail.com (N.N.); bakhen@dut.ac.za (B.N.)

**Keywords:** Zero Trust, communication, navigation, surveillance, cybersecurity, access control, network security, Zero Trust Architecture, security, implementation, policy, micro-segmentation, assets, AI, IoT, ICAO, regulations, ADS-B, GNSS

## Abstract

The increasing interconnectivity and digital transformation of Communication, Navigation, and Surveillance (CNS) systems have expanded their attack surface, rendering traditional perimeter-based security models inadequate for protecting these critical infrastructures. Zero Trust Architecture (ZTA), founded on the principle of “never trust, always verify,” offers a paradigm shift towards continuous, context-aware security. This paper presents a literature review investigating the application of ZTA principles to secure modern CNS ecosystems, following the guidelines of the International Civil Aviation Organization (ICAO) through its Cybersecurity Strategy and Plan. We analyze the alignment of ZTA core tenets—such as least-privilege access, micro-segmentation, and continuous authentication—with the unique operational requirements of CNS systems. This paper also presents a cybersecurity framework, under development within the Future Communications Digital Infrastructure (FCDI) project of the SESAR JU program, which aims to assist CNS stakeholders in collaboratively identifying cybersecurity threats within their scope of responsibility. The review critically examines implementation challenges for specific CNS subsystems: secure aeronautical communications (e.g., LDACS), resilient PNT (Positioning, Navigation, and Timing) services, and integrated surveillance networks (e.g., ADS-B, multilateration). Furthermore, we identify and evaluate domain-specific challenges, including integration with legacy avionics and ground systems, managing stringent latency and reliability constraints, and protecting against sophisticated threats targeting supply chains and data fusion processes. By synthesizing current research and practical deployment insights, this review aims to provide a foundational reference for aerospace engineers, cybersecurity specialists, and policymakers, offering a roadmap to enhance the cyber-resilience of vital CNS infrastructure in an era of evolving digital threats.

## 1. Introduction

The integration levels that have been increasing as a result of Information and Communication Technology (ICT) tools into the mechanical equipment that is used in the aviation industry have introduced concerns regarding the current cybersecurity protection frameworks’ resilience.

Considering the needs of the sector, security compliance in terms of cybersecurity poses as another challenge in the evolution of the aviation industry through the deployment of smart airports and e-enabled airport infrastructures.

The strategic global position held by the aviation industry acts as a bridge between nations. The resilience of CNS infrastructures to support operational integrity is of vital importance as minor oversights and errors may result in major damages, e.g., fatalities, loss of workforce critical information, loss of stakeholders, theft of credentials, and intellectual property.

Interconnectivity of systems, together with the adoption of the newest technologies like cloud computing, Internet of Things (IoT), and artificial intelligence (AI), has permitted seamless communication and innovation. This connectivity has been the core enabler of digital transformation; however, it has also led to the increased cybersecurity risks that include cyber-attacks and data breaches [1].

To overcome these complex threats, the traditional perimeter security approaches need to be enhanced [2]. Zero trust has come to the rescue by offering enhanced data protection, defense, and visibility against fast-paced cyber threats through continuous verification and authentication of all entities in the network [2].

With the benefits of ZTA mentioned above, it has emerged as a paradigm-shifting approach to cybersecurity against the traditional security models that trust internal network traffic and users [3]. ZT assumes that the network is under constant attack and that the network entities could be compromised. ZTA operates on a principle of ‘never trust, always verify,’ to ensure continuous verification of all devices, applications, and users while enforcing authentication, intense access controls, authentication, and its mechanisms [4]. Continuous monitoring, policy enforcement, and least privileges are core tenets of the Zero Trust Architecture. Fine-tunes access controls are implemented where users are only granted minimum rights associated with the level of access that will only enable them to access and execute only certain tasks [5]. This assists with reducing the leverage of the attack and containing the risks associated with external breaches and insider threats.

**Figure 1:**
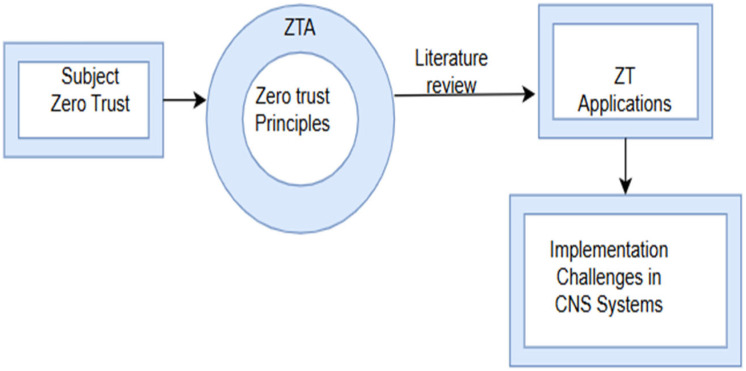
Workflow of the study.

Since the Zero Trust Architecture is still in its preliminary stages within the aviation sector, the focus is still on the framework itself, algorithms, trust evaluations, access controls, and identity authentication [6]. Hence, this survey aims to provide the current state with regard to Zero Trust Architecture development. Figure 1 below illustrates the workflow of this research article.

## 2. Background

Network security has been relying on the perimeter security model as the dominant model for information security. This model depicts that the users inside the corporate network perimeter can be trusted, and anyone who is outside is not trusted and regarded as a potential threat to the network. For decades, this approach of trust has been used to determine what resources an asset/subject can access [7].

Zero trust security surfaced and challenged this idea as the cybersecurity landscape continued to incline and took advantage of intrinsic network trust. It has taken the stance of “never trust, always verify” by carefully examining all entities in the network (users, devices, applications), irrespective of whether it is inside or outside of the network [8].

The concept of Zero Trust has been there in cybersecurity before it was even called “Zero Trust.” Its early breakthrough was the establishment of “black core” by Defense Information Systems Agency (DISA) and the Department of Defense to try and enhance enterprise security by shifting the focus from perimeter-based protection to one that focused on individual transactions [9]. After that, the foundation of Zero Trust was laid, and there were different evolutions from there for de-parameterization that improved into the larger concept of Zero Trust. Zero trust then became the concept that described numerous cybersecurity solutions that shifted from intrinsic trust to evaluation of individual transactions [10].

Civil Aviation’s air and ground systems are becoming highly connected and integrated, supporting future Air Traffic Management (ATM) optimization needs. Future Communication, Navigation, and Surveillance (CNS) systems will enable the digital revolution by integrating previously separate services and infrastructures. The evolving cybersecurity threat landscape and the increasing potential for attack propagation between systems making current isolated security measures ineffective. To provide interoperable solutions without gaps in protection, cybersecurity threats to CNS systems can no longer be addressed separately for ground and aircraft. As a result, CNS stakeholders require assistance in collaboratively establishing and maintaining an effective cybersecurity posture [11].

## 3. Zero Trust Principles

The Zero Trust model is based on principles that set it apart from the traditional security models. The Zero Trust security tenets involve a proactive approach in the network by constantly assuming that the network is under continuous threat from cyber-criminals and users with malicious intentions [12]. It also emphasizes that the security risks are not only emanating from external sources, but they can also emanate from compromised devices and users with malicious intent, which then concludes that the geographical location cannot be used as the deciding factor to grant trust. Above and beyond physical or virtual location, there must be authentication and authorization procedures for all users and nodes to permit access to the network resources. Secondly, the least privilege access ZT approach limits permissions to only those that users and applications require to perform their tasks as defined in the policies and rules for each user and application [12]. Zero Trust networks also incorporate micro segmentation, which divides the network into smaller segments to reduce the attack surface while ensuring that the access controls are different among the segments [13]. This ensures that the traffic between segments is controlled and there is no compromised user account or devices upon detection of an attack. ZT security also emphasizes multi-factor authentication to verify and authenticate all users and devices that require access to the network resources. This ensures that the password is not the only form of authentication to gain access but requires users to present more than one form of authentication evidence for resource access, thus enhancing authentication security. Encryption is regarded as one of ZTA’s cornerstones due to its crucial role in securing the stationary data, in-transit, or during processing. It transforms sensitive data into a normal format by replacing key names with identifiers that can be decoded to extract the information. This ensures that even if the attackers can gain access to the network, they will not be able to easily access sensitive data. Continuous monitoring is adopted to ensure that the policies are dynamic, and real-time data analysis from diverse sources is conducted to monitor the behavior of users, devices, and network traffic to adapt to the changing cybersecurity landscape [14].

### Taxonomy of ZT Network Features

**Figure 2:**
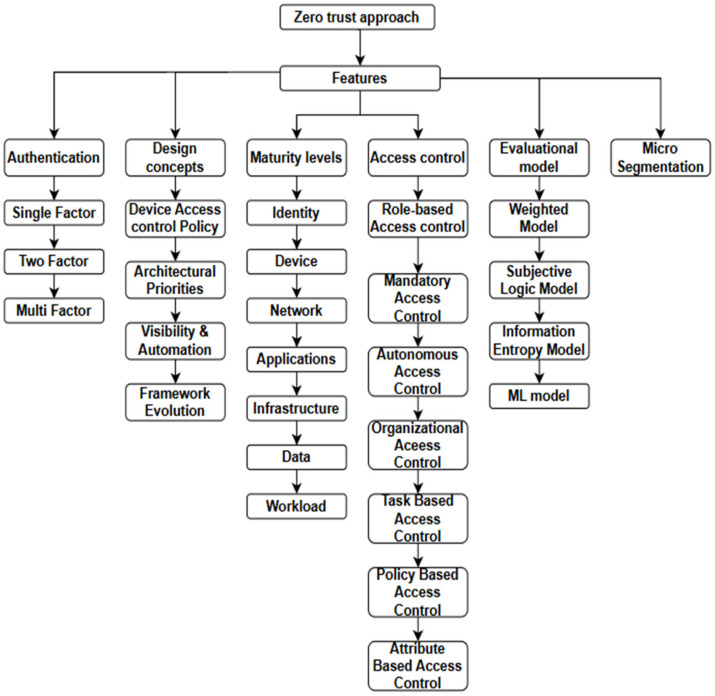
Taxonomy of ZT network features.

Figure 2 below illustrates the classification of ZT network features.

The authentication feature is responsible for validating users’ credentials in order to differentiate between legitimate users and actors with bad intentions attempting to gain illegal access to the network. To ensure only legitimate users are able to access the network, strong authentication procedures are required [15].

ZT network’s design policies are the ones that guide the Zero Trust network’s operation and implementation. It includes Architectural policies, device access policies, automation, and frameworks [16].

ZT maturity levels allow for tracking progress towards full Zero Trust model implementation. Conventional level indicates a serious lack in ZT implementation, whereas the advanced level denotes that there are steps taken to partially implement the ZT model; finally, the optimal level indicates the full implementation of Zero Trust with complete automation in place [17].

Access control is a critical ZTA prerequisite that distinguishes the privileges of users or applications and limits access based on those privileges. Its primary aim is to protect the information, programs, and components by ensuring that the activities performed on them are regulated [18].

Continuous evaluation critically evaluates and examines the access requests from the auxiliary platform that collects data so it can generate trust values. These trust values serve as the authorization mechanism that enables dynamic and fine-tuned trust assessment [19].

Micro-segmentation splits the network into small segments that have their own set of access control policies and security to limit the impact of the breach.

## 4. Zero Trust Architecture

**Figure 3:**
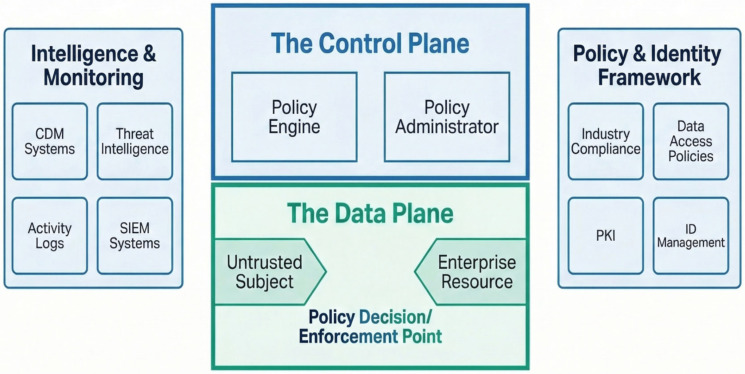
Zero Trust Architecture.

Zero trust is a cybersecurity model that prioritizes protection by implementing ZT principles within a business network infrastructure. Its focus is mainly on the interaction of components, access control, and workflow planning to enhance security measures in a network [20]. The deployment of ZTA within an organization consists of various logical components that are interconnected and can be implemented as an in-house or cloud-based service. Figure 3 shows the main components and their interlinkage as displayed on the NIST’s ZTA model.

The policy decision point is a crucial component that determines the resource access based on company policies using the trust algorithm. It consists of two elements: Policy Administrator (PA) and Policy Engine (PE). A PE decides whether a certain subject should be granted access to a resource by trust algorithm application, company policies, and external data sources. PA component executes the PE decisions into action, it further initiates and terminates sessions between subjects and resources, also generates the authentication tokens that are session-specific, and configures the Policy Enforcement point as per the need. PEP has the flexibility to operate as a single component or be divided amongst the client and resource sides to extend the trust zone outside the PEP [21].

Besides the core components, both internal and external data sources provide the input to policy rules for a PE to use TA about access decisions [22]. The enterprise security infrastructure incorporates several other interconnected components. Those components of a ZT network are:

1. The Continuous Diagnostics and Mitigation (CDM), which focuses on maintaining the asset states and is responsible for all software components and upgrade configurations. It ensures the correctness of OS patches and software integrity.
2. Industry compliance ensures the regulatory framework and policy rules are enforced to comply with the industry standards.
3. Threat intelligence provides information on vulnerabilities, attacks, and malware from internal and external sources to the policy engine.
4. Activity logs provide insights about network congestion, operations of assets, and all other activities to the enterprise data system security status.
5. Data access policy establishes the rules and policies that govern access to resources.
6. Enterprise public key infrastructure generates and logs certificates that are potentially integrated with the global PKI ecosystem for requisitions, subjects, and services.
7. Lastly, Security information and event management collects security data for analysis so it can fine-tune policies and issue real-time alerts on possible attacks on enterprise assets.

## 5. Literature Review

### 5.1. ZTA

Syed et al. have explored various authentication and access controls with the context of ZTA, his focus was on encryption, security automation, micro-segmentation, and also covered the software-based perimeters and their challenges.

A comprehensive survey by Naeem Firdous Syed et al. explored the Zero Trust security framework, providing a brief understanding of its principles, evolution, and applications. It explores important ZTA elements and defines their responsibilities in enhancing security, such as identity management, continuous monitoring, and micro-segmentation. The research highlights the benefits and challenges of ZTA adoption in the evolving cyberthreat domain while evaluating its effect in various sectors. The paper provides a summary of organizations that are facing the inclining cyber threats and highlights the reasons why the corporation should align its security strategy with this innovative security approach.

Cao et al. centered his research around deploying AI techniques to automate the adoption of ZTA, highlighting the challenges and benefits that come with orchestrating the model. Yan and Wang composed key technologies that are associated with Zero Trust, together with their applications in various scenarios, to qualify the benefits of adopting the Zero Trust approach [23]. Dayna et al. suggested a Zero Trust model specifically for cloud data center networks, that model uses the combination of identity management, automated threat response, and authentication based on packets that dynamically control all eight various network trust levels of the model [24].

Pedro Assuncao proposed a Zero Trust Architecture that challenges static credentials, utilizes multifaceted verification, and keeps track of all devices and congestion in the network [25]. Lukaseder T proposed a Zero Trust Architecture framework that seeks to address security issues in the Moodley e-learning platform to demonstrate performance gains of the web server. Further tests are still required to evaluate the ZTA model’s non-functional performance [26]. Pitman et al. proposed the implementation of Zero Trust principles directly to data objects other than access pathways [27].

### 5.2. Zero Trust Applications

Shan Li et al. delved into the convergence of the ZTA model and the Internet of Things (IoT), focusing on future industry applications. The authors stress the importance of considering security in the IoT domain, as it has increased the adoption by businesses, and emphasize the need to integrate the Zero Trust concepts. The literature review stresses the importance of ways in which Zero Trust is allowing safe IoT installations in industries like manufacturing, smart cities, and healthcare. The practical cases were used in the study to provide valuable insights into data security and preservation in the evolving IoT landscape.

Keyvan Ramezanpour et al., delved into the domain of Zero Trust Architecture within the 5G and emerging 6G networks, focusing on principles and challenges in conjunction with incorporating ML techniques together with Open Radio Access Network (ORAN) technologies. The analysis highlighted how 5G and 6G networks are dominating the telecoms sector while promoting Zero Trust approaches to guard against emerging cybersecurity risks in these networks. The research also highlighted the importance of network segmentation, verifications of identity and access controls, and its longstanding relevance. Machine learning techniques play a crucial role in enhancing network security by employing real-time adaptive responses, intelligent threat detection, and anomaly detection, as explored by the literature.

Sultana et al. presented a ZT concept-based safe medical image allocation using blockchain technology. This technology ensures the enhanced protection of sensitive medical data. The complexity and efficiency consequences of blending these technologies need to be critically examined, which opens room for further research in this field.

Additionally, a web biometrics-based user authentication and access control system was explored in [28], demonstrating response times of around 120 milliseconds. However, this system suffers from scalability and flexibility limitations, risks false access blocking due to biometric thresholds, and raises privacy concerns.

### 5.3. ZTA in Communication, Navigation, and Surveillance (CNS) Systems

A growing body of research has explored ZTA applications in aviation and critical infrastructureAnother study introduced “DistriTrust,” a ZTA-based authentication algorithm that distributes trust across multiple policy decision points (PDPs) using threshold signatures [28]. 

In [29], the authors developed ZT design patterns for enhancing aviation system security and privacy. Using a simulated Unmanned Aerial Vehicle (UAV) surveillance system, they demonstrated that applying ZT assurance patterns significantly improved resilience against cyber threats.

Compared to single-signature approaches, DistriTrust reduces authentication latency and improves reliability, making it a promising candidate for CNS integration [29].

Despite these advances, challenges remain. The distributed nature of CNS deployments increases the attack surface, requiring frameworks that can balance operational complexity with rigorous security. This highlights the need for further development of ZTA-based models tailored specifically to aviation, ensuring both enhanced security and compliance with evolving international regulatory standards [30].

Ukwandu et al. suggest a deception solution for the early detection of breaches in critical infrastructures, as current infrastructures prove to be ineffective. With the threats that are continuing to increase in the civil aviation industry, focusing on stealing data for political and financial gains that may cause long-term business disruptions and possible loss of lives [30].

A.A Elmarady and K. Rahouna (2021) investigate cybersecurity in civil aviation with a focus on developing and applying a comprehensive risk assessment framework. The study analyses vulnerabilities across communication, navigation and surveillance systems, and satellite-based technologies. It proposes a structured methodology for identifying, evaluating, and mitigating cyber risks in aviation environments [31].

Kagalwalla and Churi emphasized the emerging challenges in provisioning cybersecurity in the civil aviation sector due to the rapid increase in the deployment of ICT technologies like cloud computing and storage, machine learning, and the Internet of Things (IoT), along with the inherent vulnerabilities that go along with them [32].

Cyber Risk International cites that the high level of connectivity, combined with digital transformation, segmentation, complexity, and the surge in global travel that enforced the recent solutions as the main contenders to the rise in cybersecurity challenges.

Kagalwalla and Churi cite again that the lack of staff training, resources, and funds is part of the contributing factors, as they become insider threats without even knowing, and may expose critical information to cyber-criminals [32].

Moreover, Duchamp and Korhani highlight the increase in air travelers and expanding/building airports, and complexities of new aircraft that directly increase the complexities of CNS systems to complement airborne equipment, stimulating an increase in civil aviation cyber-attacks [33].

Duchamp, Korhani and Bayram, Abeyratne and Kessler et al. have the view that the high reliance on IT systems in the day-to-day operations of the industry has enabled improvements in the complexity of air navigation, airborne, and communication systems intensify the surface of cyber-attacks [33].

Paganini confirms that the attackers who can successfully gain access to aviation systems and disrupt operations are the ones with a broad understanding of the system’s functions, citing that the attack on an entire aviation system is not easy and straightforward [34].

Haass, Capezzuto and Sampigethaya cite those technologies, such as cloud computing, IoT, Wireless Fidelity (Wi-Fi), Global Positioning System (GPS), Internet, and open-source systems, which have been central in aviation operations optimization, reducing response time and costs through enhanced interoperability. These systems can, however, be targeted remotely as a result of their vulnerabilities [35].

Tedeschi investigated the satellite-based communications threats and possible solutions in 2022, which are also the infrastructures used within the aviation industry. Researchers alluded that there is a potential that these systems may be exploited by attackers, taking advantage of system design through back-doors, insecure data transmission protocols, weak, hard-coded credentials, and weak algorithms utilized for encryption [36].

The 2017 report by Biesecker already stipulated that a group of researchers had managed to successfully gain access remotely and hack Boeing 757 systems via radio frequency communication in a setting that is non-laboratory.

Authors in [37] proposed a Zero Trust Avionics Systems (ZTAS) to mitigate the risks for cyber-attacks and intrusions in safety-critical flight control systems. They reiterated that a well-defined approaches for simultaneously designing functionality and ZT cybersecurity are required. These approaches should maintain functionality while ensuring the system’s confidentiality and integrity. The application of Zero Trust across various components was then explored, including sensor components, actuator components, computing hardware components, software algorithm components, communication network components, and validation and verification procedures.

In summary, the high reliance of avionics systems as part of the evolution to meet current and future demands of air traffic management has resulted in high exposure to cyber-attacks. Legacy CNS equipment issues and fragmentation significantly escalate the challenges, as they were not designed to withstand cyber-crime.

## 6. Zero Trust Model Implementation

**Figure 4:**
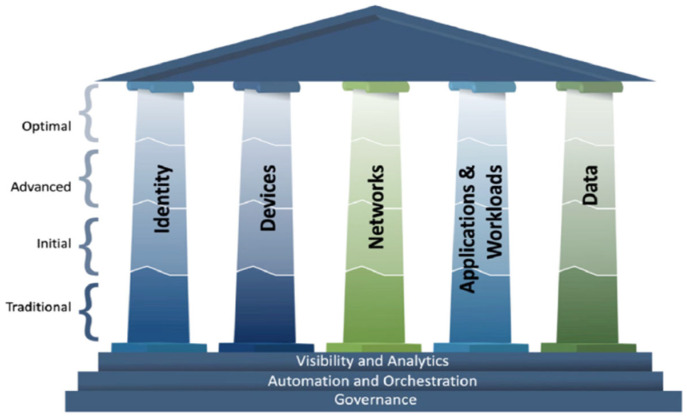
ZT security pillars [21].

The implementation of Zero Trust involves deploying measures and protocols that promote the notion of not trusting any device or entity in a network. The implementation process includes the pillars of the ZT model for robust security protection. Figure 4 shows the five pillars of the Zero Trust model divided into devices, identity, applications, data, and network environment. Each pillar has common components for automation and orchestration, visibility & analysis, and governance.

Identity describes the necessity of unique features of entities or agency users for establishing secure access controls. Various hardware assets, IoT interconnected devices, and mobile phones connect with the network to enable safe and efficient communication and data exchange. Moreover, applications and workloads that are either located on-premises or in the cloud rely on this network infrastructure to function. The interrelated systems allow for data flow, which is the core fundamental to agency operations. Furthermore, visibility and analytics bring insights to organization-wide events, which guide decisions and proactive security approach actions. Automation and orchestration regulate the security response actions and, therefore, enhance the resilience and efficiency of the model. Lastly, governance enforces cybersecurity policies across the organization and ensures ZT principles and requirements are adhered to.

### Zero Trust Maturity Model

Most companies, when adopting ZT principles and concept, utilize the existing infrastructure; implementing ZTA fully may require additional capabilities so it can fulfill its promise fully. It is not always possible to transition to a full ZTA in one go; instead, the transition can be in stages depending on enterprise priorities. The National Institute of Standards and Technology (NIST) has publicized the seven tenets of ZTMM as outlined in the ZT guide. These classifications are as follows:

i. Securing every communication across the network
ii. Considering all data sources and computing services as resources
iii. Granting access per session for individual enterprise resources
iv. Access control for resources governed by dynamic policies.
v. Enforcement of dynamic authentication and authorization for resource access
vi. The integrity and security standing of organizational assets are monitored and assessed intensively.
vii. Enhancing overall security posture through data collection on assets, communications, and infrastructure.

The NIST acknowledges that full implementation of all tenets may not be achievable within a stipulated strategy, but ZTMM can be used to track progress towards full implementation.

**Figure 5:**
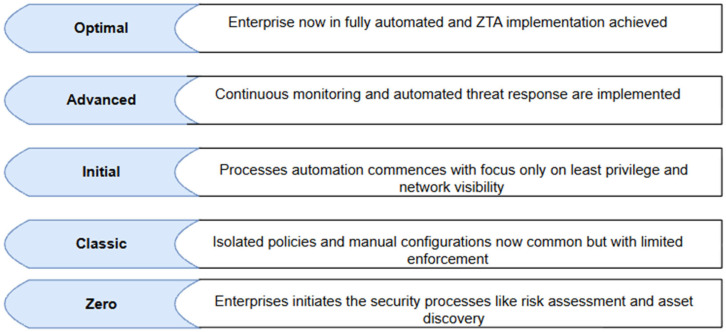
ZTMM stages.

The journey towards Zero Trust full implementation unfolds in five stages, as shown in Figure 5 below.

## 7. Zero Trust Implementation in CNS Systems

### 7.1. Industrial Practices and ICAO Standardization

Digitization has brought significant cybersecurity challenges, mainly because legacy trust models are highly incompatible with IP-based, open, and broadcast-dependent architectures. In order to address vulnerabilities in subsystems such as GNSS, ADS-B, and concepts in line with Zero Trust Architecture are currently being explored.

For Zero Trust implementation in CNS systems, the following documentation shall be adhered to as they provide regulatory baseline that any ZTA framework must comply with.

- Annex 10 Volume III—Communication Systems: Part I addresses Aeronautical Telecommunication network (ATN), Communication Systems, and VHF Digital Link (VDL) Modes 2, 3, and 4. These standards are the ones that define communication paths that ZTA policy enforcement points must secure.
- Global Navigation Satellite System (GNSS) Manual (DOC 9849) and Performance Based Navigation (DOC 9613): These documents provide guidance on GNSS and PBN implementation and operation. For ZTA applied to navigation systems, the authentication and integrity requirements for navigational data must align with the provisions outlined in these manuals.
- Manual on Secondary Surveillance Radar (SSR) Systems (DOC 9684) and Manual on Mode S Specific Services (DOC 9688): These documents outline surveillance data formats and exchange protocols [38,39]. Micro-segmentation strategies for ZTA must operate within these defined protocols.

Recent developments in Zero Trust Architecture (ZTA) within aviation systems must be comprehended in alignment with global regulatory frameworks as defined by the International Civil Aviation Organization (ICAO). Identity management, resilience, and secure information sharing are highlighted in ICAO cybersecurity guidelines, including the Aviation Cybersecurity Strategy and Trust Frameworks that closely align with ZTA.

Furthermore, the integration of ZTA mechanisms into the CNS environment is influenced by the stringent criteria imposed on safety-critical systems by avionics certification and system assurance standards like DO-178C and DO-254 [40].

Though the International Civil Aviation Organization (ICAO) has not explicitly standardized ZTA, its recent requirements on redundancy, resilience, and integrity assurance implicitly align with ZTA concepts. CNS systems are used across various nations. Implementing a ZTA framework requires collaboration across different countries’ regulatory bodies (e.g., FAA, EASA) and approval from international bodies like ICAO and EUROCAE WG-72.

### 7.2. Summary of ICAO Cyber Security Strategy and Action Plan

Based on the most recent information from the International Civil Aviation Organization (ICAO), the Aviation Cybersecurity Strategy is built around a comprehensive framework of seven pillars, and the Cybersecurity Action Plan translates that strategy into concrete tasks and priority actions for implementation.

Table 1 summarizes the key components of the Strategy and Action Plan:

The Seven Pillars of the Strategy

- International Cooperation
- Governance
- Policy and legislation
- Information sharing
- Incident Management and Emergency Planning
- Capacity Building and Cyber Hygiene
- Security by Design

#### 7.2.1. The Cybersecurity Action Plan (CyAP)

The Cybersecurity Action Plan (CyAP) is the key implementation instrument for ICAO’s aviation cybersecurity strategy, turning the high-level seven-pillar framework into practical activities using an organized collaborative approach. The CyAP divides each of the seven pillars into 32 Priority Actions, which are then broken down into fifty-one specific tasks aimed at managing the multifaceted nature of cyber threats to the aviation industry. Critically so, the plan outlines the duties and responsibilities of three primary stakeholder groups—ICAO, individual States, and industry partners to ensure that accountability is spread, and actions are coordinated across the global aviation ecosystem. Recognizing that the cybersecurity landscape is constantly changing, the CyAP is kept as a living document; the first was published in 2020, followed by a second updated edition in 2022, reflecting the need for continuous refinement to address emerging threats and technological advancements.

#### 7.2.2. Recent Developments and Current Status

The framework continues to evolve to address emerging risks like drone interference and Artificial Intelligence (AI). The following key updates from the latest ICAO Assembly (2025):

Mandatory Implementation: States are now being urged to implement the Strategy and Action Plan, which includes appointing national aviation cybersecurity authorities and developing national plans.

New Challenges: ICAO is actively working on new threats such as GNSS jamming/spoofing, the risks posed by weaponized drones, and the cybersecurity implications of AI in aviation.

Institutional Support: A Cybersecurity Panel (CYSECP) and Trust Framework Panel (TFP) have been established to develop technical standards and manage the security interchange of digital data across the aviation industry.

#### 7.2.3. Mapping ICAO Strategy and ZTA

The ICAO cybersecurity plan does not explicitly mandate Zero Trust Architecture, but it simply provides the governance, trust, and security principles necessary for ZTA implementation. ICAO can be considered as an enabling framework, whereas ZTA is a technical implementation model for achieving those strategic objectives in aviation systems, as stipulated in Table 2 below.

#### 7.2.4. Regional Variations in Aviation Cybersecurity Approaches

The key finding of this review is the variance in regional approaches to aviation cybersecurity and Zero Trust adoption, which has significant implications for international CNS interoperability.

The European SESAR Joint, as outlined in Table 3 below, is the most advanced regional initiative that specifically incorporates Zero Trust concepts into CNS systems. This solution aims to define processes and governance for a unified, global approach to cybersecurity in CNS, including threat scenarios, security requirements, and mitigation measures based on ZTA principles [41].

### 7.3. CNS Subsystems ZTA Implementation Considerations

Global Navigation Satellite System (GNSS): navigation data is a crucial ZTA enforcement point due to GNSS’s susceptibility to spoofing and jamming attacks. Future designs might incorporate behavior-based anomaly detection, continuous GNSS signal authentication, with policy engines dynamically adjusting position confidence based on sensor fusion and signal integrity metrics [42,43].

Automatic Dependent Surveillance-Broadcast (ADS-B): ADS-B’s broadcast nature, together with its known lack of authentication and integrity protection, presents both vulnerability and a testbed for ZTA principles. In a Zero Trust paradigm, ADS-B data from untrusted sources (e.g., crowdsourced receivers) would be assigned lower confidence scores and cross-validation with other surveillance sources (radar, multilateration) before being fed into ATM decision systems [44].

L-band Digital Aeronautical Communications Systems (LDACS): As an emerging air-ground communication technology, LDACS offers unique ZTA deployment opportunities. The IP-based structures of LDACS allow for software-defined perimeters and micro-segmentation, but the aeronautical channel imposes tight latency and reliability constraints that must be quantified in the security framework development [45].

Table 4 below outlines the current projects that are in motion to formulate an effective framework for the implementation of ZTA in CNS systems.

### 7.4. Zero Trust Architecture for CNS Systems

To implement the four guiding principles in a distributed avionics system, designers should consider all the building blocks of current cyber-physical systems. Zero Trust for CNS systems design and development incorporates several layers and components, such as sensors, actuators, processing units (HW), algorithms (SW), a communication network, and verification methods.

**Figure 6:**
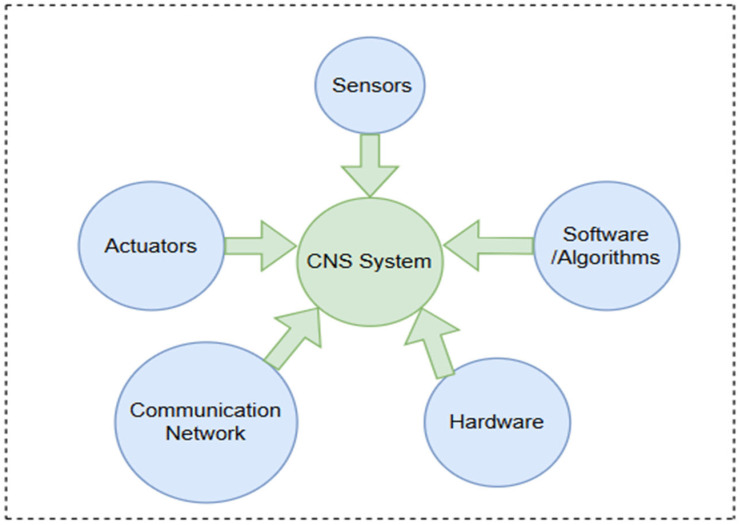
Zero Trust CNS system architecture.

Figure 6 illustrates the key techniques currently focused on, which are classified across different system components.

#### 7.4.1. Zero Trust in Sensor Components

The Zero Trust CNS systems design in Figure 6 typically incorporates sensor components for the system to be trustworthy based on their physical activity. The messages received via a data communication bus are mostly assumed to be authentic. However, this current approach poses significant security risks if cyber-criminals manage to breach the sensors.

The sensors are one critical component that is incorporated in CNS systems, which implies that data from them is inherently trusted. This presents several limitations, because firstly, there is no verification of device identification, meaning that any counterfeit or unverified sensor can be connected to the system. There is also no integrity protection or message authentication, which leaves sensor outputs vulnerable to replay attacks and spoofing. The cross-sensor validation before the data is processed is also not considered yet. In CNS systems, sensors such as air data, GPS, and radars are susceptible to manipulation and spoofing, and without behavioral validation, they can feed misleading data into navigation and control algorithms.

a. Online authentication

Fostering secure collaboration between sensors and platforms, similar to how TLS and HTTPS protect web transactions, could be an effective protection. This model enables components to recognize and authenticate each other, establish secure connections, and measure firmware. This technique could protect intellectual property in the semiconductor industry and prevent physical assaults and unauthorized firmware updates. The platform’s protocol challenges new sensor components connected to the system. For example, if a drone’s camera, GPS receiver, or Inertial Measurement Unit (IMU) is changed, then the platform would check its authenticity using this protocol with public key cryptography certificates.

b. Fault-Tolerant Redundancy

The dependability of Integrated Circuits (ICs) is becoming a key concern due to technical difficulties introduced by diminishing nodes. Reliability and performance are complex issues due to sensitivity to external factors such as radiation-related effects (from cosmic rays or radioactive decay), electromigration, high temperatures, process variation, and transistor aging. To implement a Zero Trust approach, it is imperative that robust and fault-resistant systems that can withstand manufacturing faults and transient errors are designed.

Designing fault-tolerant systems has been practiced for decades, particularly in aviation, space exploration, and healthcare. This strategy enables a system to function in a degraded mode, rather than failing completely due to a malfunctioning component. Several techniques for fault tolerance and recovery mechanisms are discussed as follows:

Triple Modular Redundancy (TMR) involves running the same code simultaneously across three redundant modules (System on Chips) on a platform. The results are processed by a majority-voting aggregator into a single output. If one of the three modules malfunctions or fails, then the other two can correct the fault and continue operating without any interruption. However, this technique increases power, size, and weight by three times, making it unsuitable for autonomous machines like drones. This is because the efficiency of a drone is determined by its available battery capacity.

Another technique is Dual Modular Redundancy (DMR), which uses two redundant modules to execute the same code simultaneously. A voting aggregator identifies potential malfunctions and failures. This technique conserves power, size, and weight but cannot recover from faults.

Checkpointing with the roll-back technique is another method: instead of comparing the outputs of each module at every instruction, it just captures periodic snapshots of the system in a good state of execution. So, when a fault occurs, the system can revert to a recent snapshot. Implementing such mechanisms for real-time systems may be difficult and can even impair functionality. Aircraft may crash due to delays caused by roll-back operations while they are attempting to recover from a fault. The roll-forward approach allows both modules to execute their jobs speculatively. A third module, temporarily powered up, can determine which of the two original modules is malfunctioning. After identifying the fault, the faulty module will roll forward to match the state of the other module, allowing recovery from the fault.

c. Continuous Digital Twin Validation

The Zero Trust concept emphasizes continuous verification and monitoring for sensor components in a digital twin framework. Continuous verification verifies a sensor’s identification and status at each interaction, not only during initial authentication. The system validates the sensor’s identity using cryptographic methods and ensures its software is up-to-date, uncompromised, and running within expected parameters. Continuous Monitoring involves observing sensor behavior and data in real-time to detect anomalies. The system scrutinizes sensor behavior, data, and network interactions for any deviations from normal patterns to discover anomalies using machine learning algorithms. Any detected anomalies trigger real-time alerts and appropriate response mechanisms. A digital twin environment allows for modeling and simulation of potential issues, experimenting with various responses, and optimization of verification and monitoring processes without affecting the actual physical system.

#### 7.4.2. Zero Trust on Actuator Components

Actuators, like sensors, are key components and are very crucial in systems. They perform physical actions in a system based on the inputs they receive, and this makes them targets for cyber-attacks aimed at disrupting the operation of the system. Most ZT approaches for sensors can also be applied to actuators. Since actuators are the components that change the system’s states, the Runtime Assurance concept can be related to ZT for the actuator components.

a. Runtime Assurance

RTA, ZT, and actuator components work together to ensure safety and security in actuator-controlled systems. The ZT security concept requires verification of all interactions with actuator components, including continuous authentication, data encryption, and network segmentation. Meanwhile, RTA is a constant, continuous process ensuring the correct and safe operation of the system. The process involves monitoring actuator statuses, ensuring command safety, and reacting to anomalies in real time, such as shutting down the actuator, activating redundancy systems, or alerting operators.

#### 7.4.3. Zero Trust on Computing Hardware Components

Once a piece of hardware is manufactured and shipped to the user, it can be challenging to update or modify it to guard against new security threats that may emerge. Below are a few ZT strategies and principles that can help to mitigate this issue.

a. Secure by design

This technique entails developing secure hardware from the ground up. Security is a primary consideration during hardware design and development, and not an afterthought. Designing hardware with security in mind involves lowering the attack surface, implementing strong access controls, and protecting critical data through encryption. The secure by design principle advocates proactive vulnerability remediation to prevent future threats from exploiting them.

b. Security Through Obscurity

Adding obscurity can improve hardware security, but it is not a stand-alone strategy. The goal is to make the hardware system difficult for attackers to understand or predict. Proprietary protocols, scrambled memory layouts, and obscured firmware can help achieve this. Although this does not make the system immune to attacks, it does increase the barrier for possible attackers, thus stalling and adding an extra layer of security.

c. Hardware-Based Security Features

Certain security features can be implemented directly into the hardware. Secure boot mechanisms, hardware random number generation for stronger encryption, and cryptographic key storage modules are examples of security measures. Hardware-based features can provide strong defense against software-based attacks and other various threats.

d. Redundancy and Resilience

Building redundancy and resilience into hardware ensures continuous operation, even if a component of the system is compromised. Redundant components can take over if primary components fail or are compromised. Error-checking and correction techniques can maintain data integrity. Designing fail-safe modes helps prevent catastrophic failures, while automated recovery techniques can restore operations after disruptions. These factors improve the system’s resilience and recovery from attacks.

e. Physical Security

Ensuring the physical security of hardware is another key consideration. This prevents attackers from tampering with or directly accessing the hardware to obtain sensitive data. Security measures can range from simple locks to advanced methods like tamper-evident seals, transparent casing, and self-destruction mechanisms that delete sensitive data upon detection of tampering.

f. Lifecycle Management

Although hardware tends to be more static than software, it can nonetheless be modified. Hardware components can be changed or replaced over time to address evolving threats. The hardware lifecycle management procedure must be secure and strong to avoid unauthorized alterations. In some cases, “firmware” updates can modify the hardware’s operation. In others, it might involve a secure, physical replacement process. Maintaining hardware systems over time ensures maximum security against evolving threats.

#### 7.4.4. Zero Trust on Software Algorithm Components

Compared to hardware, software can be patched and updated remotely as new threats are identified. Below are some key ZT principles and strategies for ensuring software security over time:

a. Principle of Least Privilege

The principle of least privilege is a central element of Zero Trust. Software components should only have sufficient permissions to execute their task. This technique reduces the risk of component compromise by requiring consistent verification of identity and authorization, reinforcing Zero Trust policy. Data in a computer system can be in three states: in transit (moving across networks), at rest (stored), or in use (for processing or computation).

Confidential computing protects data and code, ensuring correct computation in applications. Implementing Zero Trust Architectures can be streamlined by ensuring data confidentiality, data integrity, and code integrity. This eliminates uncertainties from operating systems, hypervisors, and other applications. Traditionally, highly privileged operating systems or hypervisors have had unrestricted access to application memory, which poses a vulnerability. Historically, industry efforts have concentrated on strengthening these components to prevent attacks. Confidential computing involves assigning resource management to operating systems or hypervisors and restricting their access to application memory, transforming the current model. This shifts the trust dependency from operating systems or hypervisors to the hardware, thus reducing the Trusted Computing Base (TCB) and further enhancing security [46].

b. Secure Coding Practices

Implementing secure coding practices is crucial in a Zero Trust environment. Developers should be aware that any system, including internal components, can be compromised. To ensure security, code should be built defensively, with the assumption that any input can be a potential attack vector. To reduce security concerns, it is important to validate input, handle errors securely, and conduct regular code reviews.

c. Software Patches and Updates

Maintaining the latest patches and updates is crucial in a Zero Trust system. Patches and updates often address known vulnerabilities that could be exploited by attackers. A compromised component could undermine the whole Zero Trust strategy, so ensuring timeous updates for all components is of utmost importance.

#### 7.4.5. Zero Trust on Communication Network Components

The security of the communication network is of paramount importance as they are critical element in any system, especially considering a Zero Trust Model. Below are the main points related to securing these components:

a. Network Segmentation

Network Segmentation holds a significant value in the Zero Trust Framework. Network Segmentation involves dividing the network into smaller networks that function independently. This method aims to prevent lateral movement within the network, particularly for possible invaders to CNS systems. If an attacker gains access to one portion of the network, then segmentation prevents intrusion from spreading further and affecting other parts, especially because ground CNS systems are highly interconnected. As a result, if a device in one segment is compromised, the threat is confined to that specific segment, minimizing the overall impact on the network [47].

b. End-to-end Communication Encryption

The Zero Trust concept emphasizes encryption for all network traffic. Encrypting data during transit prevents hostile actors from manipulating or exploiting intercepted data. Encryption should apply to all data, not only sensitive information. Even seemingly harmless data may provide valuable insight to a skilled attacker [48].

c. Intrusion Detection and Prevention Systems (IDPS)

Zero trust emphasizes the importance of intrusion detection and prevention technologies. These systems monitor network traffic to detect malicious activities and policy violations. When an IDPS detects a danger, it can take defensive actions like blocking or reducing network traffic to protect network integrity [49].

d. Software Defined Perimeter (SDP)

A software-defined perimeter contributes to implementing a Zero Trust model across network components. An SDP creates an invisible network to outsiders. Access to network resources is controlled based on user identification, device, and context, rather than the network location alone. This approach adheres to the Zero Trust Principles and does not trust any user or device by default, regardless of their position within or outside the network.

e. Micro Segmentation

Micro-segmentation, a finer level of network segmentation, is particularly beneficial in cloud networks. Micro-segmentation separates workload and application components into independent segments. This method reduces the attack surface and prevents emerging risks within a network. This approach protects the entire system by isolating each part.

f. Network Resilience

A Zero Trust strategy requires designing a network that can resist attacks and failures and quickly recover from them. To ensure system reliability, redundancy, effective failover mechanisms, and rapid incident response are necessary. A resilient network is capable of limiting damage from breaches and recovering quickly and effectively, rather than only resisting attacks [49].

#### 7.4.6. Verification and Validation (V&V) Procedures

Incorporating Zero Trust principles into system engineering verification and validation procedures can enhance the reliability and security of a system. Here is how Zero Trust can be applied to these processes:

a. Verification

The verification phase should ensure that avionics systems are designed to adhere to Zero Trust principles from the outset. This includes ensuring strong access restrictions, network segmentation, and strict data protection standards are in place in avionics systems. Check critical components like sensors and actuators to ensure they function on the “least privilege” principle, eliminating extraneous permissions that could expose them to potential risks.

b. Validation

Validation tests the system to ensure it meets the end user demands and performs as per the specifications. To implement Zero Trust, security measures must be tested to confirm their effectiveness. For example, conduct a penetration test to prevent access to the system, or simulate a compromised device to ensure the system detects and responds effectively.

c. Continual Verification

CNS systems are dynamic and require continuous monitoring. Continuous verification is a key component of implementing Zero Trust. Implementing real-time assurance systems with constant monitoring can help ensure system integrity. Anomalies in sensor readings or unexpected behaviors in actuators must trigger rapid alerts and responses [37].

d. Audits and Documentation

Logging and analyzing all CNS system actions is crucial for detecting abnormal behaviors. The trail helps in identifying and understanding potential threats. Effective documentation of V&V processes ensures a clear and traceable record of system behavior and responses to security events.

e. Feedback Loop

CNS systems, like any other Zero Trust implementations, should prioritize continuous improvement. Regular monitoring, testing, and incident responses can provide valuable insight for system design and operation. If a vulnerability is identified in the CNS subsystem, then corrective measures should be implemented and re-verified to ensure the issue is fully remedied.

(a) Software/Algorithms

The computational logic and background processes are trusted by default. The systems do not represent secure boot, runtime integrity monitoring, firmware signing, and partitioning mechanisms. This introduces risks such as unauthorized parameter changes, firmware modifications, and exploitation of vulnerabilities in algorithm implementations.

In the absence of memory isolation and runtime verification, compromised software could generate unsafe actuator commands, and navigation solutions may be manipulated. In safety-critical CNS systems, software must not only meet minimum functional requirements but also extend to maintaining integrity to guard against intentional tampering.

(b) Hardware

Hardware is another trusted contributor to the CNS systems without any visibility of security controls. There is little to no hardware root of trust, cryptographic key storage, secure elements, and supply chain protection mechanisms. This presents the risks like counterfeit circuit boards, hardware bugs, and bitstream replacements. Since avionics systems have a long-life span, supply chain and lifecycle integrity are critical. The security architecture assumes trust without any demonstration of how that trust is established and maintained.

(c) Communication Network

The communication network interconnects CNS systems with external components and other avionics systems without authentication, encryption, and segmentation in place. Traditional CNS communications buses do not include additional protective layers and native security features, which makes them susceptible to spoofing, message injection, replay attacks, and denial-of-service conditions. The communication network allows for seamless communication without restrictions like strict identity verification and least-privilege communication policies between system elements.

### 7.5. Generic Challenges with ZTA Implementation in CNS Systems Across Air Navigation Service Providers (ANSPs)

Implementing the Zero Trust principles in avionics systems presents a different set of challenges that need attentive strategic and planning responses. While the benefits of Zero Trust are significant, to maximize its benefits and full adoption, the challenges need to be carefully navigated.

The biggest challenge with the adoption of Zero Trust Architecture in CNS systems is the fact that, amongst the three disciplines, there are legacy systems that are still used and were only compatible with traditional security models, but with the increase in modern cybersecurity threats, those models are no longer effective.

When the CNS data is shared across centers, the external service providers are involved as they are the ones with the infrastructure to transfer the information at a correct bit rate and in real-time, since timestamping is the most crucial measure for the validity of information. That poses challenges, especially with encryption, as the collaborative decision-making (CDM) system needs to be in place and effective so that there can be that surety that the data shared is not vulnerable to cyber-attacks (e.g., interception of voice between the air traffic controller and pilot).

Another challenge with the full implementation of ZTA in CNS is the design that enables continuous patching and updating mechanisms throughout the lifespan of equipment, which is usually between 10 and 15 years. The sad reality is that the original equipment manufacturers usually stop supporting their products after a few years and recommend the purchase of their new products, and in aviation, that is not practically possible.

**Below are other key barriers to Zero Trust implementation.**

**Cultural Barriers and Organizational Resistance**

The transition from the traditional security approaches to the Zero Trust framework requires a lot of cultural **shifts** in the aviation sector. This sector is promoting safety culture, and time is also a crucial part in all tasks conducted, hence why there may be resistance to adopting continuous verification and privilege access principles of zero trust. The new change management strategies, transparent communication, and comprehensive training need to be implemented to adopt a Zero Trust framework.

**Technical Complexity and Integration**

CNS systems can be complex as a standalone system because of how they are integrated with each other. Integrating Zero Trust on top of that can be very technically challenging and would require extensive resources. Surveillance technologies, navigation, and diverse communication legacy systems may present compatibility challenges. Maintaining operational functionality is a key priority to ensure safety in aviation, so there is a need for careful planning and implementation in stages to ensure seamless interconnectivity amongst CNS and Zero Trust components.

**Balancing Security and Usability**

The other challenge with Zero Trust adoption is finding the balance between Zero Trust-intensive security measures and user experience. Continuous verification and least privilege access policies may lead to delays in system restoration and also frustrate the user, which can lead to operational inefficiencies. To overcome this, aviation entities should streamline authentication processes, intuitive interfaces to optimize workflows in ensuring that security enhancements do not hinder operations.

**Data Privacy and Regulatory Compliance**

In a highly regulated industry like aviation, continuous verification and access controls may raise data privacy concerns. The companies will need to ensure compliance with ICAO and GDPR mandates while implementing robust security measures. This also necessitates the understanding of legal requirements and data governance practices.

**Financial considerations**

To implement Zero Trust, there is a need for significant investment in technology, maintenance, and staffing. Aviation organizations will have to allocate resources for upgrading security infrastructure, deploying monitoring tools, and personnel training. Balancing the costs and benefits will require a thorough cost–benefit analysis and strategic resource allocation.

**Organizational scalability**

Strategies used for the implementation of Zero Trust shall also accommodate the scalability and growth of organizations in the aviation sector. To ensure Zero Trust architecture is still effective even after the organization expands and evolves, careful architectural planning and adaptability to changing operational needs are required.

## 8. Materials and Methods

### 8.1. Using Machine Learning Techniques for Detecting Anomalies in ADS-B Data

In this work, the authors address significant cybersecurity vulnerabilities in the Automatic Dependent Surveillance-Broadcast (ADS-B) protocol, which is essential for real-time aircraft tracking but lacks native security mechanisms. Recognizing the increasing restrictions on air control systems as air traffic volumes rise, the authors offer a machine learning-based framework for detecting and correcting abnormalities in ADS-B time series, with a focus on low-altitude traffic.

The methodological contribution is organized around four genuine attack scenarios: spoofing, saturation, replay, and interpolated ghosts, each addressed by a unique algorithmic approach. To detect spoofing attacks, the authors use a Convolutional Neural Network (CNN) classifier trained to identify trajectory shapes that are inconsistent with the declared aircraft type, exploiting spatial patterns to distinguish between authentic and fabricated positions.

To mitigate saturation attacks, which overload the system with excessive data, a Long Short-Term Memory (LSTM) model is utilized to estimate future aircraft positions; differences between predicted and observed trajectories serve as indicators for anomalous conditions, allowing corrective action. Replay attacks are identified through a hash-based method that checks the temporal uniqueness and integrity of transmitted messages.

Finally, interpolated ghost attacks in which false aircraft are injected artificially using smooth trajectory interpolation. This work has been packaged in a Python 3.14.0 library easily utilized to ensure anomaly detection in ADS-B data from any source.

ADS-B protocol is used to monitor aircraft. It is commonly used to secure air traffic, avoid collisions, or provide help to aircraft in distress. As the name indicates, it is a broadcast protocol, which means that the plane’s transponders publicly disseminate the messages. ADS-B messages can then be decoded by ground receivers or received by nearby aircraft. ADS-B messages are generally broadcast every second, and their Type Code identifies the type of information it contains. An ADS-B message normally contains the following information:

- Timestamp: when the message was emitted in seconds
- ICAO24: the 24-bit identity of the transponder
- Callsign: the registration code of the aircraft
- Track: orientation in degrees. 0° is North, 90° is east.
- Groundspeed: horizontal speed in knots
- Position: latitude and longitude
- Squawk: a code between 0000 and 7777 that indicates the status of the aircraft or could be used by ATC to ease aircraft identification and traffic management.
- Altitude: barometric, in feet
- Alert: a Boolean that indicates whether the aircraft is in distress or not
- Vertical rate: vertical speed, in feet per minute.
- Geometric Altitude: calculated from GPS, in feet.
- Spi: indicates whether the Special Position Indicator (SPI) has been set or not.

### 8.2. Dataset

The dataset was built using OpenSky Network, a free and open source of historical ADS-B data. The OpenSky was chosen because most other sources, like FlightRadar24, require registration and provide incomplete data. The data was gathered between 2022 and 2023 in Toulouse, France, a busy area with a huge airport, military bases, and aero clubs. Only low-altitude traffic under 10,000 feet was maintained. The raw data was separated into files for each aircraft. Flights with poor quality caused by coverage gaps, signal challenges, or transponder errors were eliminated. To ensure high-quality for machine learning, flights were only maintained if they had less than 10% missing data and lasted at least 15 min.

The authors developed a Python library to easily plug into any system. The Flask web server was provided on GitHub to perform anomaly detection for the ADS-B visualizer tool. The authors also stipulated that the results can be repeated or reproduced with the code available on GitHub.

### 8.3. Spoofing Attacks

#### 8.3.1. Scenario

The first attack scenario focuses on spoofing attacks. Spoofing is the use of another aircraft’s identification to conceal oneself or obtain special authorization. The scenario took place at Toulouse during the 2023 Rugby World Cup. A drone might potentially target the stadium by spoofing the identity of an emergency helicopter. This would allow the drone to fly over sensitive areas without raising suspicion. Toulouse was chosen for its intensive traffic, as it is an international airport and has a high volume of low-altitude traffic. Additionally, the city has two hospitals with heliports, resulting in significant traffic of emergency helicopters.

Initially, the attack was imagined with a drone as they are a topical subject. However, due to a lack of drone ADS-B data, this plan was set aside to focus on traditional aircraft, as such an attack could also be carried out by a tourist aircraft.

#### 8.3.2. Method

To detect spoofing attacks, the model was trained to identify aircraft that are not flying as expected based on their type. Different aircraft have different flight patterns: commercial planes fly smooth and linear, light planes can be chaotic with loops, and helicopters often fly straight at low altitude or hover.

The model was trained to classify flights into three categories: commercial planes (only during takeoff and landing), light planes, and helicopters. Each flight in the database was labeled as one of the three categories.

The model utilizes a sliding window technique. Each window comprises 128 time steps (about 2 min of flight) and includes nine features such as position, speed, altitude, and timestamp. Because the model predicts each message, the final decision is made by averaging the thirty-two highest-confidence predictions.

The best results were obtained using the Adam optimizer with a learning rate of 0.0002 and Mean Squared Error loss instead of the standard classification loss. To boost accuracy, the training data was deliberately imbalanced, with label ratios changing dynamically between training rounds based on model performance. This resulted in a slight accuracy gain of 0.24 points on the evaluation dataset.

#### 8.3.3. Results

To verify the effectiveness of the model and compare it with other architectures, other models were trained and evaluated, as seen in Table 5. The authors of [11] investigated various time series classification techniques on twelve multivariate datasets, which influenced some of these models. Several convolutional and recurrent models, including a reservoir computing model from ReservoirPy. Reservoir models gained prominence in recent studies, prompting researchers to test them. Reservoir models differ from other models as only the output layer is trained, leaving the input and reservoir connections untouched after initialization. The number of parameters varies significantly between models. The Transformer is a shallow model with less than 9000 parameters, while MCDNN has more than 6,000,000 parameters.

Prediction quality is measured using accuracy as a parameter. Real-time classification requires an indication of the inference time for each message. The results for the spoofing attack scenario are summarized in Table 5. The models are rated according to their performance, with CNN and ResNet at the top and Encoder offering the lowest accuracy. The highest accuracy and inference time are indicated in bold. Convolution-based models provide the highest accuracy. Recurrent architectures, such as LSTM, perform well but have a very long execution time.

The hand-made CNN model is equally accurate as the ResNet model. ResNet is deeper than CNN and uses a convolution layer with a kernel size of one in its skip connections to match the size of the tensor before and after each block.

ResNet is about twice as low as the CNN model. Such a difference will highly reduce the possibilities for real-time predictions. The main point of uncertainty is between tourist aircraft and helicopters. This is due to similarities in flight behavior between emergency helicopters and tourist aircraft. Some recreational aircraft fly in a straight line at speeds nearly the same as those of helicopters. The limited number of classes prevents spoofing detection between identical aircraft, such as commercial planes and private jets, as they are represented by the same class. The datasets used already have sufficient data to train a model with more precise labels.

Adding too many classes reduces the model’s accuracy significantly. Increasing class granularity might lead to confusion between close classes. If the model encounters an aircraft type that does not fit in any of the three classes, it will likely assign the trajectory to the closest matching class. Drones are typically classified as helicopters and do not cause anomalies due to their registry code being included in the system.

### 8.4. Saturation Attacks

#### 8.4.1. Scenario

Saturation attacks are a type of flooding attack in which ghost aircraft are injected around a real aircraft. The injected ghost aircraft gradually diverges, usually following the path of the real aircraft. The attack utilizes the same ICAO24 as the real flight, making it difficult to distinguish one from the other. This attack targets a specific flight, making air traffic control impossible for that specific aircraft.

#### 8.4.2. Method

Solving saturation attacks requires identifying actual aircraft among a large number of ghosts. The genuine aircraft has the most natural trajectory. Solving saturation attacks involves ranking ghosts based on their abnormal or inconsistent trajectory.

The proposed method is to train a deep learning model to predict aircraft coordinates based on their current path. The model is trained with the Adam and a learning rate of 0.0003. The MSE loss is applied to train the model. It is crucial not to confuse the error rate with the loss function, since the error rate is a metric that is used to evaluate model error in meters. Once trained, the model error rate serves as an indicator of abnormality. The model error is calculated using the Harvesine distance between the ADS-B message and the prediction. The model processes ADS-B time data in sliding windows of 32 messages each. It allows us to constantly check if the flight goes well. As a result, a saturation can be detected easily as the error will reach an abnormal peak.

Once saturation attacks are detected, the algorithm waits and computes an error value for each ghost aircraft. After five predictions are made, the mean error is calculated, and the ghosts are ranked. The aircraft with the lowest error rate is considered the legitimate one. The model includes several optimizations to improve predictions. Trajectory windows are normalized using relative coordinates: latitude and longitude are transformed so the last message is at 0° North, 90° east, and the track is normalized to always face north. However, this normalization initially caused issues because most training targets ended up in the same spot, making the model behave like a constant function. To fix this, the model was trained only on trajectories with sufficient curvature. Curvature is measured by taking four points along the trajectory window: at the start, one-third, two-thirds, and the end. The curvature is calculated by adding two angles formed by these points. If the total curvature is greater than 5 degrees, the trajectory is included in the training dataset. In addition, the distance to the next message was provided as input to improve model prediction.

Providing the distance to the next message as input helps the model focus on predicting the angle of the next message. This approach avoids oversimplifying the prediction task, which is important because maintaining complexity maximizes the contrast between prediction errors of ghost aircraft and legitimate aircraft.

The optimal prediction horizon was also investigated, which is the temporal distance between the input and the predicted message. It was found that predicting at a horizon of t+5 gave the highest accuracy for detecting the true aircraft. Finding the horizon with the lowest loss is not always the best option, even if it is counterintuitive. Predicting at t+1 yields a very good error (around 3.1 m) but does not perform well against saturation attacks. A short horizon makes the model too sensitive to unusual trajectories. The model accurately predicts the ghost’s path, even when it behaves abnormally. During an attack, the prediction error is not significant enough to distinguish between the ghost path and the aircraft’s actual errors. Training a model on a far horizon can lead to inaccurate long-term predictions.

#### 8.4.3. Results

Multiple models were tested to handle saturation attacks, evaluating them on 33 attacks involving 15 aircraft with ghost trajectories diverging at various angles.

Three models were compared:

##### LSTM

The LSTM model used for processing saturation is a residual hand-made model with a convolution layer at the front to aid pattern extraction. The output of the final LSTM cell is used directly as the prediction.

##### Transformer

It is a Pytorch-based transformer model adapted for time series regression tasks.

##### Math

It is an algorithmic model used as a baseline to demonstrate the benefit of deep learning. It predicts the aircraft’s next position using spherical coordinates based on estimated track and speed from a window of sixteen timestamps.

The mean speed is used to analyze an aircraft’s speed. Testing linear regression and derivative approaches has produced unreliable findings due to abnormalities in flight.

Finally, the estimated track was calculated using linear regression on the aircraft’s angular velocity. Table 6 compares the performance of the various models. Accuracy is based on the rank of legitimate aircraft along the ghost. For 100% accuracy, the legitimate aircraft is always ranked first, while 0% indicates it is always ranked last. The error is the average distance between predictions and the actual aircraft in meters. The “perfect” column represents the percentage of saturation attacks where the legitimate aircraft has been ranked first.

The LSTM is the most accurate, achieving 97.82%. It successfully resolved 69.7% of saturation attacks in the evaluation dataset. Perfectly solving saturation attacks in our dataset requires the model to distinguish the ghost target diverging at −1° and 1° from the actual aircraft. Although it functions well on clean flights, particularly on straight trajectories, missing ADS-B data during saturation can cause issues.

### 8.5. Replay Attacks

#### 8.5.1. Scenario

Replay attacks are another type of flooding attack. This attack involves re-emitting an ADS-B stream of recording from a previous flight. These attacks generate ghost flights that are non-existent and are perfect trajectories by nature. Replay attacks could have a substantial impact on frequent airlines. For example, in Southern France, taxi-helicopters operate between Nice, Cannes, and Monaco. Helicopter flight paths are repetitive as all aircraft follow the same trajectory. Repeated replay attacks at regular intervals could hinder air traffic controllers from distinguishing between the actual and ghost aircraft. Such an attack would disrupt traffic and compromise air traffic control safety.

#### 8.5.2. Method

The study was initially based on deep learning, but the researchers quickly discovered that it is not well-suited for detecting replay attacks because ghost trajectories appear flawless. Instead, detection requires comparing current flights against a historical database to see if the same trajectory has occurred before.

To detect replays before the flight ends, the comparison is made on small trajectory segments rather than entire flights. Traditional methods like DTW distance are too slow and unreliable when working with a large historical database. Therefore, hash algorithms were chosen as the preferred solution.

The hash algorithm was developed inspired by the Shazam algorithm for time series recognition. It compares the flight segments of 32-time steps. However, unlike audio recognition, aircraft trajectories can be easily rotated, translated, or scaled by an attacker, so the hash function must be invariant to these transformations.

To achieve this, the hash function is based only on latitude and longitude. The trajectory is first converted into a fingerprint that records only the series of slight right and left turns made by the aircraft. This fingerprint remains the same even if the trajectory is translated, rotated, or scaled. The fingerprint is represented as a list of zeros and ones (right and left turns) and is converted into a hash by computing its decimal representation according to Equation (1) below.

(1) $$B\left(f\right)=\sum f_{i}\times 2^{i}$$

where $f_{i}$ represents the $i^{th}$ element of the binary fingerprint.

This prevents collisions in the hash function, making it impossible for two fingerprints to yield the same hash. This formula does not distribute close values as a standard hash function does, but this is not relevant to our case. However, this method has a flaw in that it generates different hashes for mirrored trajectories. A mirrored trajectory will generate the exact opposite fingerprint. One of the solutions is to reverse any hash starting with “1” to its reciprocal. Hence, 001…010 will remain the same, but 110…101 will be changed into 001…010 using Equation (2) below.

(2) $$\left\{H(f)=\;\right.\left\{\begin{array}{c} B(f)\;\;\;\;\;\;\;\;\;\;\;\;\;\;\;\;\;\;\;\;\;\;\;\;\;\;\;\;\;\;\;\;\;\;\;\;\;if\;B(f)<2^{\left(n-1\right)}\\ {(2}^{n}-1)-B(f)\;\;\;\;\;\;\;\;\;\;\;\;\;\;\;\;\;\;\;\;\;\;\;\;\;\;\;\;\;\;\;\;\;otherwise\;\;\;\;\;\;\;\;\;\;\;\;\;\;\;\;\;\;\;\;\;\;\;\;\;\;\;\; \end{array}\right.$$

where f is the fingerprint and *n* is its length.

#### 8.5.3. Results

For detecting replay attacks, the researchers discovered that 32-time step segments provided an optimal balance. Shorter segments (16 steps) resulted in too many duplicate fingerprints, while longer segments (64 steps) raised computing demands.

The algorithm worked exceptionally well, detecting every replay in the database and catching most altered trajectories, including those that were shifted, rotated, scaled, or mirrored. Detection accuracy depends on how much the trajectory was altered, but this is not a major concern because when the algorithm fails, the trajectory becomes so distorted that it appears unnatural and is likely to be detected by other methods.

One of the algorithm’s key strengths is that it never yields false positives because it is impossible for thirty-two consecutive hashes from different flights to perfectly match. Detection speed is fast and does not depend on the size of the database, generating the hash table can be time-consuming and requires significant storage for large databases.

The primary constraint is that the algorithm can only detect a replay if the original flight exists in the database. Since many flights occur near cities and airports, the database required for complete coverage might be rather enormous.

### 8.6. Interpolated Ghost Flights

#### 8.6.1. Scenario

Interpolated ghosts are the final type of fake aircraft that the other detection methods were unable to identify. These ghosts are constructed using mathematical models, so their paths appear smooth and natural, with no noticeable anomalies.

Most interpolated ghosts are easy to identify because they have unknown call signs, follow unnatural trajectories, or appear in areas with no real traffic. However, a well-crafted ghost can be far more difficult to identify without radar. Such a ghost can appear normal, evading both the saturation detection and replay detection models.

Despite being much more difficult to detect, these ghosts pose a serious threat. Like replay attacks, they can be used to flood congested air lanes with bogus aircraft, potentially disrupting real air traffic operations.

#### 8.6.2. Method

Interpolated ghosts are difficult to identify because they are mathematically generated and lack the natural noise found in real ADS-B data. While an attacker can add artificial noise to make them appear genuine, the noise may still contain imperfections that provide evidence.

To detect these ghosts, researchers trained a classifier model to distinguish between interpolated and real ADS-B trajectories. The model employs sliding windows with 32-time steps, striking a balance between accuracy and processing speed. It makes its first prediction after approximately 32 s (32 ADS-B messages) and then updates the prediction with each new message. When the moving average over the last 128 predictions indicates that the flight is interpolated by more than 90%, an anomaly is detected.

The model was trained with the Mean Squared Error (MSE) loss and the Adam optimizer at a learning rate of 0.0003. To generate training data, the researchers took actual flights and deleted 90% of the ADS-B messages before filling in the gaps with a Bezier curve-based interpolation algorithm method. The algorithm estimates the aircraft’s vectors on both sides of the gap and determines the point where they intersect. The vectors are then normalized at two-thirds of the distance between the gap edges and the intersection point to generate a smooth, realistic-looking curve.

#### 8.6.3. Results

The researchers evaluated and compared several techniques for detecting interpolated ghost flights. The models were tested on a dataset of 34 flights, 17 normal and 17 interpolated. Almost all models performed very well. Table 7 presents the results using multiple metrics:

Accuracy (flight level): Percentage of flights correctly classified as either normal or interpolated.

Accuracy per message: Percentage of correct predictions made per individual ADS-B message; this metric was used to rank the models.

Precision: Proportion of predicted positives that are actually true positives.

Recall: Proportion of actual positives correctly identified.

F1-Score: Harmonic mean of precision and recall, balancing both metrics.

ROC-AUC: Probability that the classifier ranks a randomly chosen positive higher than a randomly chosen negative one.

FPR (False Positive Rate): Proportion of actual negatives incorrectly classified as positive.

The authors note that the flight-level accuracy was generally 100%, meaning the models correctly classified every flight.

### 8.7. Discussions

The authors presented four machine learning algorithms, each designed to detect a specific ADS-B anomaly: spoofing, flooding, replay attacks, and interpolated ghost trajectories. While specialized, these algorithms can be utilized to detect other anomalies, such as virtual trajectory modifications. A comparative evaluation showed that convolutional neural network (CNN)-based models offer the best balance between handling complex detection tasks and computational efficiency. The algorithms were designed to be lightweight, successfully processing a dozen simultaneous flights at 64 times real-time speed on generic hardware. Unsupervised approaches, such as GAN-based discriminators for generic anomaly detection, were recommended as a future research focus. The future scope will also include assessing model resilience against adversarial attacks, which present a known vulnerability for deep learning-based detection systems [50].

## 9. Conclusions

Zero Trust security principles provide a comprehensive method for protecting complex systems from emerging threats in the digital landscape. While originally developed for network security, these principles are also applicable across different subsystems and components of complex cyber-physical systems, including CNS systems.

In this paper, we explored the current state of ZTA implementation across regions and the projects that are in motion to address the cybersecurity threats in CNS infrastructure. Since aviation is a highly regulated sector, the recommended practices are adopted from ICAO by nations through local Civil Aviation Authorities (CAA) and cascaded down to all stakeholders, including ANSP, airlines, and designers. The crucial documents were highlighted with precise operational guidance for consideration to ensure alignment while developing security frameworks.

This study examines the application of Zero Trust across various CNS components, including sensor or actuator, computing hardware, software algorithms, communication network, and verification and validation procedures. The goal is to safeguard each component of the system, which collectively contributes to the overall system’s security and robustness.

This review also investigated the effectiveness of specialized machine learning algorithms in identifying ADS-B abnormalities, focusing on spoofing, saturation, replays, and interpolated ghost attacks. The findings by the authors show that, while these models are optimized for specific attack vectors, their architecture allows for more advanced trajectory adjustments. Comparative research shows that CNNs provide a better balance of performance and computational efficiency for these complex tasks.

We also explored multifaceted challenges and strategic imperatives of deploying Zero Trust Architecture (ZTA) in the aviation industry’s Communications, Navigation, and Surveillance (CNS) systems. While the transition away from perimeter-based security is important for combating modern cyber threats, the research reveals that full adoption is severely limited by the presence of legacy infrastructure, 15-year equipment lifecycles, and the “support gap” left by original equipment manufacturers. Furthermore, the findings show a fundamental contradiction between the stringent criteria of continuous verification and the operational necessity for real-time, low-latency data exchange in flight-critical environments. Ultimately, the review concludes that successful ZTA integration requires a collaborative approach that aligns with international regulatory bodies, with a fundamental shift in organizational culture, ensuring that enhanced security standards support, rather than hindering the industry’s core safety mission.

## Figures and Tables

**Table 1:** Summary of the key components of the Strategy and Action Plan.

Feature	Description
Official Strategy	A high-level framework built on seven pillars to guide global cooperation and policy.
Action Plan (CyAP)	A tactical roadmap published by ICAO that breaks the Strategy down into 32 Priority Actions and fifty-one specific tasks for stakeholders.
Core Goal	To establish a harmonized and cooperative global approach to protect civil aviation from borderless cyber threats.

**Table 2:** Mapping ICAO strategy with ZTA.

ICAO Strategy Pillar	Zero Trust Architecture (ZTA) Implementation
Security by Design	ZTA mandates that security be incorporated into aviation systems from the outset rather than being added later. This is in alignment with ICAO’s push for proactive security measures.
Information Sharing	ZTA’s strict access controls directly serve the ICAO’s goal of secure information exchange. It ensures that data is only accessible to verified entities on a strict need-to-know basis, allowing for secure collaboration.
Incident Management	ZTA’s main principle is continuous monitoring of all network activity. This provides the real-time visibility required to quickly detect, contain, and manage cyber incidents, thus supporting the ICAO goal.
Capacity building	The transition to ZTA necessitates cultural and technical upskilling across the aviation workforce. ICAO’s focus on capacity building provides the necessary foundation for training workers to adopt and manage this new, complex architecture.

**Table 3:** Summary of Regional Variations in Aviation Cybersecurity Approaches.

Region	Regulatory Body	Key Cybersecurity Standards	ZTA Adoption Status	CNS-Specific Guidance
North America	FAA/RTCA	DO-326A/ED-202A (Airworthiness Security), DO-356A (Security Methods)	Emerging—referenced in industry guidance	Limited to general avionics security
Europe	EASA/EUROCAE	ED-202A, ED-203A, SESAR JU CNS cybersecurity framework	Active—SESAR Solution 0338 explicitly addresses CNS	Solution 0338 defines a collaborative framework for CNS security
Asia-Pacific	CAAC (China)	Adopting RTCA/EUROCAE standards with local adaptations	Exploratory	Limited public guidance
Middle East	GCAA (UAE)	ICAO-based with regional supplements	Early stage	Not specified

**Table 4:** Summary of CNS Subsystems ZTA implementation considerations.

Project/Subsystem	Current State of Development	Key Zero Trust Architecture (ZTA) Focus
SESAR Collaborative Cyber Security Framework for CNS	Active; basic principles observed at Technology Readiness Level (TRL) 1, with a target of reaching TRL 2 by June 2026.	Focuses on defining the processes and governance for a collaborative, global-scale security framework that incorporates Zero Trust Architectures.
EUROCONTROL “Trajectory 2030” Strategy	Announced in late 2025; serves as a strategic roadmap for modernization through 2030.	Commits to implementing a Zero Trust approach specifically for identity verification across all internal processes.
LDACS (L-band Digital Aeronautical Communications System)	Active development of an avionic prototype as part of the FCDI project to increase system maturity.	ZTA principles are integrated via the separate, overarching “Collaborative Cyber Security Framework” being developed in parallel.
ADS-B (Automatic Dependent Surveillance-Broadcast)	No specific, detailed ZTA implementation project found.	Addressed as a component within the broader Communication, Navigation, and Surveillance (CNS) framework.
**GNSS (Global Navigation Satellite Systems)**	No specific, detailed ZTA implementation project found.	Addressed as a component within the broader Communication, Navigation, and Surveillance (CNS) framework.

**Table 5:** Spoofing Models Comparison.

Model	Accuracy	Time per Message	Parameters
CNN	97.27%	1.38 ms	261,379
ResNet6	97.27%	2.44 ms	509,571
Inception6	97.15%	2.52 ms	447,619
FCN6	96.83%	1.44 ms	274,307
LSTM	96.77%	2.47 ms	396,547
MCDCNN6	96.53%	2.61 ms	6,024,243
Reservoir5	95.40%	5.24 ms	80,451
t-LeNet6	95.16%	1.46 ms	82,778
MLP6	93.18%	1.66 ms	823,003
Transformer7	92.42%	2.59 ms	8911
Encoder6	90.96%	3.22 ms	3,346,691

**Table 6:** Saturation models comparison.

Model	Accuracy	Error	Perfect	Time per Message	Parameters
LSTM	97.82%	12.8 m	69.7%	2.7 ms	396,930
Transformer	93.05%	180.6 m	18.2%	1.5 ms	172,418
Math	91.0%	95.8 m	45.5%	0.5 ms	0

**Table 7:** Interpolated Flight Models Comparison.

Model	Accuracy	Time per Message	Accuracy per Message	Parameters	Precision	Recall	F1 Score	ROC-AUC	FPR
Encoder	100.0%	2.17 ms	96.4%	3,261,441	100%	93.65%	96.72%	97.63%	0%
ResNet	100.0%	1.35 ms	95.9%	508,161	100%	92.56%	96.14%	97.39%	0%
FCN	100.0%	0.80 ms	95.7%	272,001	100%	91.94%	95.8%	97.03%	0%
Inception	100.0%	2.44 ms	95.1%	442,497	100%	91.27%	95.43%	97.16%	0%
LSTM	100.0%	3.10 ms	95.0%	396,289	100%	90.70%	95.12%	97.96%	0%
t-LeNet	100.0%	0.55 ms	94.7%	41,726	100%	90.31%	94.91%	97.06%	0%
CNN	100.0%	0.70 ms	94.4%	102,657	100%	89.61%	94.52%	97.02%	0%
MCDCNN	100.0%	5.79 ms	93.8%	3,024,377	100%	88.37%	93.82%	97.08%	0%
MLP	100.0%	0.48 ms	92.2%	630,001	100%	86.31%	92.65%	96.8%	0%
Transformer	100.0%	1.35 ms	91.77%	3121	100%	84.93%	91.85%	96.47%	0%
Reservoir	85.29%	1.88 ms	64.46%	32,001	99.4%	34.16%	51.94%	88.0%	0.26%

## Data Availability

No new data were created or analyzed in this study.
